# Cost-effectiveness of universal iron supplementation and iron-containing micronutrient powders for anemia among young children in rural Bangladesh: analysis of a randomized, placebo-controlled trial

**DOI:** 10.1093/ajcn/nqac225

**Published:** 2022-10-03

**Authors:** Edifofon Akpan, Sheikh J Hossain, Angela Devine, Sabine Braat, Mohammed I Hasan, S M Mulk Uddin Tipu, Mohammad Saiful Alam Bhuiyan, Jena D Hamadani, Beverley-Ann Biggs, Sant-Rayn Pasricha, Natalie Carvalho

**Affiliations:** Centre for Health Policy, Melbourne School of Population and Global Health, The University of Melbourne, Parkville, Victoria, Australia; International Centre for Diarrhoeal Disease Research, Maternal and Child Health Division, Bangladesh (icddr,b), Dhaka, Bangladesh; Department of Women's and Children's Health, Uppsala University, Uppsala, Sweden; Centre for Epidemiology and Biostatistics, Melbourne School of Population and Global Health, The University of Melbourne, Parkville, Victoria, Australia; Division of Global and Tropical Health, Menzies School of Health Research, Charles Darwin University, Darwin, Northern Territory, Australia; Centre for Epidemiology and Biostatistics, Melbourne School of Population and Global Health, The University of Melbourne, Parkville, Victoria, Australia; Population Health and Immunity Division, Walter and Eliza Hall Institute of Medical Research, Parkville, Victoria, Australia; Department of Medicine at the Peter Doherty Institute, The University of Melbourne, Parkville, Victoria, Australia; International Centre for Diarrhoeal Disease Research, Maternal and Child Health Division, Bangladesh (icddr,b), Dhaka, Bangladesh; International Centre for Diarrhoeal Disease Research, Maternal and Child Health Division, Bangladesh (icddr,b), Dhaka, Bangladesh; International Centre for Diarrhoeal Disease Research, Maternal and Child Health Division, Bangladesh (icddr,b), Dhaka, Bangladesh; International Centre for Diarrhoeal Disease Research, Maternal and Child Health Division, Bangladesh (icddr,b), Dhaka, Bangladesh; Department of Medicine at the Peter Doherty Institute, The University of Melbourne, Parkville, Victoria, Australia; Population Health and Immunity Division, Walter and Eliza Hall Institute of Medical Research, Parkville, Victoria, Australia; Diagnostic Hematology, The Royal Melbourne Hospital, Parkville, Victoria, Australia; Clinical Hematology, The Peter MacCallum Cancer Centre and The Royal Melbourne Hospital, Parkville, Victoria, Australia; Department of Medical Biology, The University of Melbourne, Parkville, Victoria, Australia; Centre for Health Policy, Melbourne School of Population and Global Health, The University of Melbourne, Parkville, Victoria, Australia

**Keywords:** iron supplements, micronutrient powders, cost-effectiveness, anemia, trial, children, low- and middle-income country

## Abstract

**Background:**

Universal provision of iron supplements or iron-containing multiple micronutrient powders (MNPs) is widely used to prevent anemia in young children in low- and middle-income countries. The BRISC (Benefits and Risks of Iron Interventions in Children) trial compared iron supplements and MNPs with placebo in children <2 y old in rural Bangladesh.

**Objectives:**

We aimed to assess the cost-effectiveness of iron supplements or iron-containing MNPs among young children in rural Bangladesh.

**Methods:**

We did a cost-effectiveness analysis of MNPs and iron supplements using the BRISC trial outcomes and resource use data, and programmatic data from the literature. Health care costs were assessed from a health system perspective. We calculated incremental cost-effectiveness ratios (ICERs) in terms of US$ per disability-adjusted life-year (DALY) averted. To explore uncertainty, we constructed cost-effectiveness acceptability curves using bootstrapped data over a range of cost-effectiveness thresholds. One- and 2-way sensitivity analyses tested the impact of varying key parameter values on our results.

**Results:**

Provision of MNPs was estimated to avert 0.0031 (95% CI: 0.0022, 0.0041) DALYs/child, whereas iron supplements averted 0.0039 (95% CI: 0.0030, 0.0048) DALYs/child, over 1 y compared with no intervention. Incremental mean costs were $0.75 (95% CI: 0.73, 0.77) for MNPs compared with no intervention and $0.64 ($0.62, $0.67) for iron supplements compared with no intervention. Iron supplementation dominated MNPs because it was cheaper and averted more DALYs. Iron supplementation had an ICER of $1645 ($1333, $2153) per DALY averted compared with no intervention, and had a 0% probability of being the optimal strategy at cost-effectiveness thresholds of $200 (reflecting health opportunity costs in Bangladesh) and $985 [half of gross domestic product (GDP) per capita] per DALY averted. Scenario and sensitivity analyses supported the base case findings.

**Conclusions:**

These findings do not support universal iron supplementation or micronutrient powders as a cost-effective intervention for young children in rural Bangladesh.

This trial was registered at anzctr.org.au as ACTRN1261700066038 and trialsearch.who.int as U1111-1196-1125.

See corresponding editorial on page 1193.

## Introduction

Worldwide, approximately 269 million (39.8%) children <5 y old were anemic in 2019, with 83 million of those affected residing in the South Asian region (1). Iron deficiency is an important cause of anemia (2), accounting for around half of all cases in rural Bangladesh (3) and globally (4). Because children aged 6–23 mo are particularly susceptible because of increased iron requirements needed for growth (5–7), the WHO recommends that those living where anemia prevalence in young children is ≥40% should receive daily iron supplements as syrups or drops (8). Alternatively, those living where the prevalence is ≥20% should receive home fortification of food with iron-containing multiple micronutrient powders (MNPs) (9). In rural Bangladesh, the 2012 National Micronutrients Survey estimated that 45% of all children were affected by anemia (10). Therefore, the 2 guidelines on daily iron supplements and MNPs are both applicable.

Systematic reviews of the effect of iron supplementation (11, 12) and home fortification with MNPs (11, 12) concluded that these interventions reduced the risk of anemia; however, whether they improved cognitive development and other functional outcomes in children <2 y old was uncertain (11–14). This is important because part of the rationale for universal iron interventions has been presumed benefit on cognitive development (15, 16). In addition, studies have indicated that iron interventions could increase the risk of diarrhea (17, 18). The BRISC (Benefits and Risks of Iron Interventions in Children) trial evaluated the effect of 3 mo of iron syrups or MNPs compared with placebo in 8-mo-old children in rural Bangladesh (19, 20). This trial tested the impact of iron interventions on health outcomes of interest (functional outcomes, anemia, and diarrhea), providing an opportunity to use high-quality data to inform policy on iron interventions. BRISC found that although iron interventions markedly reduced the prevalence of anemia, neither iron supplements nor MNPs improved child cognitive development.

Universal iron interventions are widely implemented. In 2019, ≥58 countries completed mass home fortification programs with support from UNICEF (21). Economic costs of delivering this mass intervention include procuring the intervention products as well as delivering these products to beneficiaries (program delivery costs) (22). To help policy makers plan interventions, it is essential to undertake cost-effectiveness analysis of these interventions to understand whether they are good value for money. Previous analyses adopting different analytic approaches have reported marked variations in the cost-effectiveness of iron supplements and MNPs (23–28). These variations in cost-effectiveness are likely associated with different assumptions made during the modeling process.

Prospectively collected data measured from a clinical trial measuring functional clinical outcomes represent an ideal opportunity for undertaking cost-effectiveness analysis, given most prior cost-effectiveness analyses included impacts on functional clinical outcomes based on observational data or assumptions. Using data from the BRISC trial, we conducted a cost-effectiveness analysis of iron supplements and MNPs for population-level control of anemia among young children in non-malaria-endemic rural Bangladesh.

## Methods

### Trial study design

The BRISC trial was a double-blinded placebo-controlled study that evaluated the impact of iron supplements and MNPs in young children (20). The trial took place in 3 administrative units (unions) of Rupganj, a rural non-malaria-endemic subdistrict (Upazila) in Narayangaj District, Bangladesh. Children were screened for potential eligibility at 8 mo. Those with hemoglobin concentrations < 8.0 g/dL, current febrile illness, severe malnutrition, history of blood transfusion, an inherited red cell disorder, or currently receiving iron-containing supplements were excluded. Children were randomly assigned to iron syrup with placebo powder, MNPs with placebo syrup, or placebo syrup and placebo powder for 3 mo (intervention period) and followed up for another 9 mo (follow-up period) (**Supplemental Figure 1**). Iron syrups used in the trial contained 12.5 mg Fe as ferrous sulfate. MNPs contained 12.5 mg Fe as ferrous fumarate, and in addition included vitamin A, vitamin C, folic acid, and zinc. These formulations met the WHO recommendations (8, 9). Trained village health workers (VHWs) visited children weekly during the intervention period and monthly during the follow-up period. This was consistent across all study arms. At the weekly visits the VHWs monitored and encouraged adherence.

The primary outcome of the study was cognitive development measured using the Bayley-III Cognitive composite score (20). Secondary outcomes included developmental (Bayley-III language and motor) and growth indexes, anemia, iron deficiency, and morbidity from infections [diarrhea (defined as ≥3 loose or liquid stools per day), respiratory infection, vomiting, and fever]. No difference in the primary outcome was observed between either active intervention and placebo in the trial (20), thus cognitive development could not be used in assessment of outcomes for the cost-effectiveness study. The subgroup analyses suggested an increased risk of diarrhea-related clinic visits in nonanemic children during the intervention period. The BRISC trial was prospectively registered at Australian and New Zealand Clinical Trials Registry (ACTRN1261700066038) and WHO International Clinical Trials Registry Platform (U1111-1196-1125). Ethical approval for the BRISC trial was granted by Melbourne Health Human Research Ethics Committee, Australia (HREC/16/MH/353; 2016.269) and the Ethical Review Committee of the International Centre for Diarrhoeal Disease Research, Bangladesh (iccdr,b).

### Health outcomes

The outcome for the cost-effectiveness analysis was disability-adjusted life-years (DALYs) over the trial duration of 12 mo. DALYs are calculated as the sum of years of life lost (YLLs) due to premature mortality and the years lost due to disability (YLDs) (29, 30). Because no difference in mortality was observed between the treatment groups in the BRISC trial (20), YLLs were not used in the DALY estimations. YLDs due to anemia and diarrhea were calculated by multiplying the duration of illness with the corresponding disability weights obtained from the Global Burden of Disease (GBD) study 2019 (31). These diseases were selected because of plausibility that iron interventions could affect them (17), the availability of disability weights for DALY estimation, and their discussion in WHO iron intervention guidelines (8, 9). For children with anemia who experienced diarrhea, the total YLDs were calculated by summing the YLDs from the 2 outcomes (**Supplemental Methods**).

Anemia was classified into WHO-defined severity levels (nonanemia, mild anemia, moderate anemia, severe anemia) using WHO thresholds based on hemoglobin concentration (32). Children <5 y of age whose hemoglobin concentration is ≥110 g/L do not have anemia. Those with hemoglobin concentration of 100–109 g/L have mild anemia, those whose hemoglobin concentration is 70–99 g/L have moderate anemia, whereas children with hemoglobin concentration < 70 g/L have severe anemia (**Supplemental Table 1**). Then, YLDs were calculated as the weighted average of disability weights between 2 time points multiplied by the duration between the time points (Supplemental Methods). For diarrhea, cases were categorized as moderate (if they were treated in outpatient settings) or severe (if they were hospitalized) with duration of illness of 6.4 and 8.4 d, respectively, based on a systematic review (33).

### Resource use and costs

For the base case analysis, costs were estimated from a health system perspective and included the direct costs of providing the iron intervention, program delivery costs, and unplanned health care use due to diarrhea (**Table 1**). Each child randomly assigned to MNPs was provided a total of 90 sachets. Each child randomly assigned to iron syrups received 15 mL weekly (195 mL over 13 wk) (19). In Bangladesh, iron syrups are available in 200-mL bottles; therefore, 1 bottle was used. It was not feasible to disentangle trial protocol–driven costs to obtain program delivery costs, so we used an estimate of US$5.80/child based on evidence from pilot programs from the Home Fortification Technical Advisory Group (HFTAG) (26, 34). Further details are available in the Supplemental Methods.

**TABLE 1:** Unit costs used in the base case analysis^1^

Component	Unit costs, 2020 US$	Source
Cost per bottle (200 mL) of iron syrup	0.63/bottle	BRISC trial team using unit process from ACME Laboratories Limited
Cost of micronutrient powders (1 box = 30 sachets)	0.018/box	BRISC trial team as procured from Renata Limited
Program delivery costs per year^2^	5.80/child	HFTAG pilot program (34)
Consultation cost for visit to study doctor/health center	1.69/visit	WHO-CHOICE database (37)
Hospitalization cost for primary hospital admissions	7.09/bed day	WHO-CHOICE database (37)
Cost of treating diarrhea with some dehydration^3^	0.31/case	DGDA Drug Database (56) using WHO treatment algorithm (36)
Cost of treating diarrhea with severe dehydration^3^	2.04/case	DGDA Drug Database (56) using WHO treatment algorithm (36)

1 BRISC, Benefits and Risks of Iron Interventions in Children; CHOICE, Choosing Interventions that are Cost-Effective; DGDA, Directorate General of Drug Administration Bangladesh; HFTAG, Home Fortification Technical Advisory Group.

2 Children with diarrhea were classified by their hydration status as severe dehydration (admitted to the hospital), some dehydration (treated at hospital but not admitted), or no dehydration (managed at home).

3 Program delivery costs of $4.50/child were reported in the year 2013. These were adjusted using a gross domestic product deflator and exchange rate to a 2020 value of $5.80.

Quantities of medications used in treating dehydration due to diarrhea were estimated using published guidelines that are followed in Bangladesh (35, 36). Briefly, children having diarrhea with “some dehydration” are managed with 2 packets of oral rehydration therapy (ORT) and 1 blister of zinc tablets in an outpatient setting. Those who have diarrhea with “severe dehydration” are admitted to hospital and given intravenous rehydration followed by ORT with zinc on discharge from hospital as described for “some dehydration.” Outpatient visits for “some dehydration” were measured as number of consultations. Inpatient stays (hospitalizations) for “severe dehydration” were measured using number of bed days. The numbers of consultations and bed days were obtained from BRISC trial data. Data on health care visits were collected weekly based on previous 7 d recall by caregivers during the 3 mo of the intervention phase. During the subsequent 9-mo postintervention phase, these data were collected monthly based on parent or caregiver recall for the previous 14 d. Therefore, resource use during the monthly follow-up period was proportionately extrapolated from 14 d to 1 mo. Unit costs per inpatient (hospital) bed day and per outpatient visit were from the WHO-CHOICE (Choosing Interventions that are Cost-Effective) database (37).

All costs were reported in 2020 US$. Costs collected in non-2020 dollars were first converted to taka of that year using World Bank–reported exchange rates (38). Costs in taka were then adjusted to 2020 value using gross domestic product (GDP) deflators reported by the World Bank (39), and then converted to 2020 US$ using World Bank–reported taka to dollar exchange rates (1 US$ = 84.9 taka) (38). Discounting was not needed because a 1-y time horizon was used.

### Analyses

We estimated the mean DALYs and costs for the 3 strategies—no intervention, MNPs, and iron supplements—over 12 mo. Costs to the health system were calculated by adding the cost of active iron agents (iron syrups or MNPs), program delivery costs, and cost of treating diarrhea. Incremental costs and DALYs averted were calculated as the difference in respective costs and DALYs between the iron interventions and no intervention. The incremental costs were divided by the difference in DALYs to derive the incremental cost-effectiveness ratio (ICER), which was interpreted as the additional cost per additional DALY averted. If a strategy cost more and averted fewer DALYs than an alternative strategy, it would be unequivocally not cost-effective regardless of the ICER (that is, dominated by the comparator) (40).

There were missing data for hemoglobin concentration and clinic visits. Missing hemoglobin data were assumed to be missing at random; that is, the missingness depends on observed data but not on unobserved values. These were imputed using multiple imputation by chained equations (MICE), stratified by treatment group (41). Forty imputations were performed because this number was at least equal to the percentage of missing data in the available cases (42). After multiple imputation, hemoglobin values were converted into anemia categories, from which DALYs were estimated using disability weights and duration of time spent in a none, mild, moderate, or severe anemic health state. The overall mean difference in DALYs between strategies was estimated as the average of estimates from each of the imputed data using Rubin's rule (43). We did not adjust for missing data on clinic visits because missing diarrhea data were expected to occur similarly across study arms (Supplemental Methods).

To estimate the uncertainty on our ICER estimates, we conducted nonparametric bootstrapping following best practices (44). Here, the sample data are used to provide an empirical estimate of the sampling distribution by repeated resampling with replacement. We used the bias-corrected and accelerated method which adjusts for skewness and bias of the sampling distribution (45, 46). Two thousand bootstrap replications were performed. Because DALYs were a function of imputed hemoglobin data, each resample of a missing data point was pooled from 40 multiply imputed data sets (46, 47). The bootstrapped data were used to construct a 95% CI. The differences in mean cost between each strategy and no intervention were plotted against the associated bootstrapped differences in mean DALYs averted on a cost-effectiveness plane.

To assess uncertainty in the decision to adopt any of the 3 strategies, we constructed a cost-effectiveness acceptability curve (CEAC) (44). For each bootstrap resample, the net monetary benefit (NMB) was calculated using the formula NMB = (−DALY × Threshold) − Cost. Within each resample and cost per DALY averted threshold, the strategy with the highest NMB was selected. Over all replications, the probability that a strategy has the highest NMB is the proportion of bootstrap replicates that yields the highest NMB. We then plotted this probability against ranges of cost per DALY averted thresholds. Two decision thresholds were used as reference in interpreting ICERs and the CEAC. These were $200 (representing an approximate average of 2 modeled health opportunity costs for Bangladesh) (48, 49) and $985 (half of GDP per capita of Bangladesh in 2020) (50).

We conducted 3 scenario analyses. In the first scenario, we used only participants with hemoglobin measurements at all time points (complete cases) for DALY calculations, instead of multiply imputed data used in the base case. In the second scenario we adopted a societal perspective instead of the health system perspective used in the base case, by incorporating household out-of-pocket costs and productivity losses. The Supplemental Methods provide details of the procedure for estimating out-of-pocket costs and productivity losses. In the third scenario, we used data from the first scenario (complete cases) and extended the estimated intervention benefits beyond the 12-mo trial follow-up period by 3 additional months (to the target population age cutoff of 23 mo). During these 3 mo, we assumed those in the active iron groups would have the same hemoglobin concentrations as they did at the end of the trial follow-up period.

A 1-way sensitivity analysis was conducted to examine the impact of input values on the ICER. Low and high values were taken from 95% uncertainty intervals when available. We did not find CIs on cost of active iron agents and program management, so we adopted a cautious approach by using a wide range: 50% in either direction. A 2-way sensitivity analysis explored the impact of simultaneously varying costs of active iron agents and program delivery. To implement this, we extracted DALYs from 5 out of the 40 imputations used in the base case (to reduce computation time). Then, we estimated the probability of cost-effectiveness at our decision thresholds of $200 and $985 over plausible combinations of the 2 cost parameters, starting from $0 with $0.10 increments. The probability was defined as a 1-sided *P* value from regression of their NMB with treatment groups as a covariate (51, 52). Separate sensitivity analyses were conducted using the 2 thresholds.

A final sensitivity analysis explored, under very optimistic scenarios, what program delivery costs will achieve ≥50% probability of cost-effectiveness. A combination of assumptions made this scenario very optimistic. First, we used only data on participants with complete cases (scenario 1). Second, we assumed the estimated intervention benefits beyond the trial follow-up period would last 3 additional months (scenario 3). Third, we used a threshold of $985/DALY averted (or half of GDP per capita of Bangladesh in 2020), which is higher than the approximate average of 2 modeled health opportunity costs for Bangladesh (48, 49).

All analyses were performed using Stata version 16.1 (StataCorp.).

## Results

### Participant characteristics

Baseline characteristics appeared similar across the treatment groups (**Table 2**). The proportion of children who consumed ≤70% of their assigned intervention was similar in the 2 active iron intervention groups (MNP arm: 70.8%; iron supplementation arm: 70.4%). Children with complete hemoglobin measurements were more likely to adhere to the intervention for ≥70% of their assigned active iron agent than were those with incomplete data: 82.2% compared with 61.0% in the MNP group and 82.2% compared with 60.1% in the iron supplementation group (**Supplemental Table 2**).

**TABLE 2:** Characteristics of BRISC trial participants by treatment group^1^

Variable	Placebo (*n* = 1100)	MNPs (*n* = 1099)	Iron supplements (*n* = 1101)
Female sex	550/1100 (50.0%)	548/1099 (49.9%)	550/1101 (50.0%)
Household wealth index			
Quintile 1: relative poorest	215/1099 (19.6%)	224/1098 (20.4%)	222/1101 (20.2%)
Quintile 3: relative middle	211/1099 (19.2%)	230/1098 (20.9%)	213/1101 (19.3%)
Quintile 5: relative wealthiest	212/1099 (19.3%)	221/1098 (20.1%)	222/1101 (20.2%)
Family care indicator^2^	13.7 ± 7.1	13.3 ± 7.1	13.2 ± 7.1
Maternal education			
No education	49/1100 (4.5%)	43/1099 (3.9%)	53/1101 (4.8%)
1–8 y	567/1100 (51.5%)	603/1099 (54.9%)	591/1101 (53.7%)
9–12 y	447/1100 (40.6%)	420/1099 (38.2%)	421/1101 (38.2%)
>12 y	37/1100 (3.4%)	33/1099 (3.0%)	36/1101 (3.3%)
Union			
Bhulta	359/1100 (32.6%)	360/1099 (32.8%)	360/1101 (32.7%)
Golakandail	387/1100 (35.2%)	387/1099 (35.2%)	390/1101 (35.4%)
Rupganj	354/1100 (32.2%)	352/1099 (32.0%)	351/1101 (31.9%)
Baseline anemia^3^	473/1067 (44.3%)	463/1057 (43.8%)	496/1073 (46.2%)
Baseline iron deficiency^4^	272/1030 (26.4%)	274/1025 (26.7%)	307/1034 (29.7%)
Baseline iron deficiency anemia^5^	188/1030 (18.3%)	182/1025 (17.8%)	225/1034 (21.8%)
70% Adherence^6^	812/1100 (73.8%)	778/1099 (70.8%)	775/1101 (70.4%)

1 Values are means ± SDs for continuous measures, and *n*/total with nonmissing data (%) for categoric measures. BRISC, Benefits and Risks of Iron Interventions in Children; MNP, multiple micronutrient powder.

2 Family care indicator total scores range from 0 to 42, with higher scores indicating more activities.

3 Anemia was defined as a hemoglobin concentration of <11 g/dL.

4 Iron deficiency was defined as a ferritin concentration of <12 μg/L, or <30 μg/L if the C-reactive protein concentration was >5 mg/L.

5 Iron deficiency anemia was defined as concurrent anemia and iron deficiency.

6 Proportion of children that consumed ≥70% of their assigned active iron agents or placebos.

### Costs and cost-effectiveness

Overall, the numbers of unplanned visits to outpatient departments and hospitalizations due to diarrhea were similar in the 3 treatment arms (**Table 3**).

**TABLE 3:** Quantities of resource use over 1 y for iron interventions compared with placebo^1^

	Placebo (*n* = 1063)		MNPs (*n* = 1053)		Iron supplements (*n* = 1072)	
Component	Mean ± SD per child	Children, *n*	Mean ± SD per child	Children, *n*	Mean ± SD per child	Children, *n*
Intervention-related						
Units of iron or MNP intervention	NA	1063	90 ± 0.00	1053	1 ± 0.00	1072
Program delivery resources	NA	1063	1 ± 0.00	1053	1 ± 0.00	1072
Health care visits^2^						
Cases of diarrhea with “some dehydration,”^3^*n*	0.148 ± 0.61	158	0.154 ± 0.59	162	0.150 ± 0.54	161
Cases of diarrhea with “severe dehydration,” *n*	0.027 ± 0.17	29	0.030 ± 0.19	32	0.024 ± 0.16	26
Inpatient bed days because of diarrhea,^4^*n*	0.038 ± 0.28	41	0.048 ± 0.38	51	0.040 ± 0.30	43

1 A small number of children had missing hemoglobin data at baseline. These children were dropped, so the sample size in this table is slightly lower than that presented in the Supplemental Methods. MNP, multiple micronutrient powder; NA, not applicable.

2 The reason for health care visits was collected for 67% of all visits during the intervention and for 95% of the trial population during the follow-up period. Visits due to diarrhea are classified using the hydration status of the child into diarrhea with severe dehydration (if the child was admitted to the hospital) or diarrhea with some dehydration (if the child was treated as an outpatient).

3 Number of cases of diarrhea with “some dehydration” was used as a proxy for number of outpatient visits, as per guidelines for diarrhea treatment followed in Bangladesh.

4 Using treatment guidelines, a child having diarrhea with “severe dehydration” is admitted to the hospital. Because a child can spend >1 night at the hospital, the total number of inpatient bed days is higher than the number of cases of diarrhea with “severe dehydration.”

The mean ± SD cost per child to the health care system was $0.62 ± $2.57 for no intervention, $8.12 ± $2.94 for MNPs, and $7.06 ± $2.58 for iron supplementation (**Table 4**). Because the program delivery costs for MNP home fortification and iron supplementation programs were assumed to be the same, differences in costs between the active iron strategies were driven by the cost of the intervention products ($1.60 for MNPs and $0.63 for syrups). No meaningful difference in out-of-pocket costs and costs due to lost productivity was observed across the 3 treatment arms (Table 4).

**TABLE 4:** Health system costs, out-of-pocket costs, and productivity loss over 1 y per child for intervention strategies^1^

	No intervention, mean ± SD	MNPs, mean ± SD	Iron supplement, mean ± SD	MNPs vs. no intervention (95% CI)^2^	Iron supplement vs. no intervention (95% CI)^2^	Iron supplement vs. MNPs (95% CI)^2^
Cost of active iron agents	0.00 ± 0.00	1.60 ± 0.00	0.63 ± 0.00	1.60 (1.60, 1.60)	0.63 (0.63, 0.63)	−0.97 (−0.97, −0.97)
Program delivery costs	0.00 ± 0.00	5.80 ± 0.00	5.80 ± 0.00	5.80 (5.80, 5.80)	5.80 (5.80, 5.80)	0.00 (0.00, 0.00)
Cost of outpatient visits	0.25 ± 1.03	0.26 ± 1.00	0.25 ± 0.91	0.01 (−0.08, 0.10)	0.00 (−0.08, 0.09)	−0.01 (−0.09, 0.07)
Cost of nonsevere diarrhea treatment^3^	0.046 ± 0.19	0.048 ± 0.18	0.047 ± 1.17	0.00 (−0.01, 0.02)	0.00 (−0.01, 0.02)	0.00 (−0.02, 0.01)
Cost of inpatient hospital stays	0.27 ± 1.95	0.34 ± 2.32	0.28 ± 2.09	0.08 (−0.11, 0.26)	0.02 (−0.15, 0.19)	−0.06 (−0.25, 0.13)
Cost of severe diarrhea treatment^3^	0.056 ± 0.34	0.062 ± 0.38	0.050 ± 0.33	0.01 (−0.02, 0.04)	−0.01 (−0.03, 0.02)	−0.01 (−0.04, 0.02)
Costs to health system (A)	0.62 ± 2.57	8.12 ± 2.94	7.06 ± 2.58	7.50 (7.27, 7.74)	6.44 (6.23, 6.66)	−1.06 (−1.30, −0.82)
Out-of-pocket costs (B)	0.503 ± 3.92	0.564 ± 3.91	0.831 ± 7.11	0.06 (−0.27, 0.39)	0.33 (−0.16, 0.82)	0.27 (−0.22, 0.76)
Costs due to lost productivity (C)	0.645 ± 3.76	0.590 ± 2.89	0.610 ± 3.19	−0.06 (−0.34, 0.23)	−0.04 (−0.33, 0.26)	0.02 (−0.24, 0.28)
Total costs (societal = A + B + C)	1.78 ± 8.04	9.27 ± 7.24	8.50 ± 9.70	7.51 (6.85, 8.16)	6.74 (5.98, 7.50)	−0.77 (−1.50, −0.04)

1 Costs are expressed in 2020 US$. MNP, multiple micronutrient powder.

2 95% CIs were obtained from 2-sample *t* test with equal variances.

3 Children with diarrhea were classified by their hydration status as severe dehydration (admitted to the hospital), some dehydration (treated at hospital but not admitted), or no dehydration (managed at home).

**Table 5** presents the mean costs and cost-effectiveness results per child for the 3 strategies. MNPs were estimated to cost $7.50 (95% CI: $7.27, $7.72) more than no intervention while averting 0.0031 (95% CI: 0.0022, 0.0041) DALYs. Iron supplementation was estimated to cost, on average, $6.44 (95% CI: $6.22, $6.67) higher than no intervention while averting 0.0039 (95% CI: 0.0030, 0.0048) DALYs. This yields an ICER of $1645 (95% CI: $1333, $2153) per DALY averted for iron supplementation compared with no intervention. Iron supplementation saved $1.06 (95% CI: $0.81, $1.28) compared with MNPs (**Supplemental Table 3**). Therefore, iron supplements dominated MNPs, and the cost-effectiveness plane for MNPs compared with iron supplementation shows this (**Supplemental Figure 2**)

**TABLE 5:** Base case cost-effectiveness results per child (with bootstrapped 95% CIs) comparing iron intervention strategies^1^

Strategy	Mean ± SD cost	Incremental cost (95% CI)	Mean ± SD DALYs	DALYs averted (95% CI)	ICER (95% CI)
No intervention	$0.62 ± $2.58	Reference	0.0075 ± 0.013	Reference	Reference
MNPs	$8.12 ± $2.94	$7.50 ($7.27, $7.72)	0.0044 ± 0.009	0.0031 (0.0022, 0.0041)	Dominated by iron supplements^2^
Iron supplements	$7.06 ± $2.58	$6.44 ($6.22, $6.67)	0.0036 ± 0.008	0.0039 (0.0030, 0.0048)	$1645 ($1333, $2153)

1 Costs and incremental costs are calculated from a health system perspective and expressed in 2020 US$. 95% CIs were calculated via bias-corrected and accelerated bootstrapping (2000 replications on multiply imputed data). DALY, disability-adjusted life-year; ICER, incremental cost-effectiveness ratio; MNP, multiple micronutrient powder.

2 The ICER comparing MNPs with no intervention ($2400; 95% CI: $1842, $3441) was not reported in the table because MNPs are dominated by iron supplements.

**FIGURE 1:**
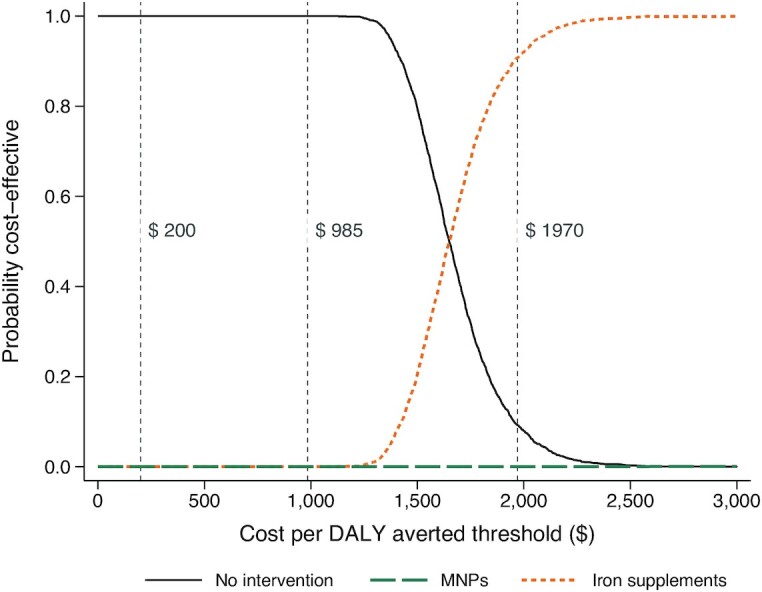
Cost-effectiveness acceptability curves showing the probability that iron supplementation or fortification with MNPs is optimal, compared with no intervention. For each decision threshold increasing from left to right on the *x* axis, the fraction of bootstrap iterations with the highest net monetary benefit is plotted on the *y* axis. The curve shows the probability that no intervention, MNPs, or iron supplements is cost-effective for an amount a decision maker is willing to pay to avert a DALY. Dashed lines indicate 3 such thresholds: $200, $985 (half the GDP per capita of Bangladesh in 2020), and $1970 (GDP per capita of Bangladesh in 2020). DALY, disability-adjusted life-year; GDP, gross domestic product; MNP, multiple micronutrient powder.

The incremental cost-effectiveness plane of bootstrapped replicates using the base case analysis showed that at $200 and $985 per DALY averted, it was unlikely that either MNPs or iron supplementation would be cost-effective (**Supplemental Figure 3**). The CEAC (**Figure 1**) showed that at thresholds of $200 and $985, neither MNPs nor iron supplements had the highest NMB in any of the 2000 bootstrap resamples (that is, 0% probability of cost-effectiveness). At a higher threshold of $1970 (GDP per capita of Bangladesh in 2020), the probability that iron supplementation is a cost-effective strategy was >95%, whereas MNPs remained not cost-effective.

### Scenario and sensitivity analyses

In the first scenario, when we restricted the analysis to only participants with complete hemoglobin data, the ICER for iron supplements compared with no intervention ($1011; 95% CI: $802, $1350 per DALY averted) was slightly lower than when using imputed values ($1645; 95% CI: $1333, $2153 per DALY averted) (**Supplemental Table 4**). In the second scenario, where we compared the societal perspective to the base case health system perspective, the cost and cost-effectiveness results were similar (Supplemental Table 4). This similarity was because there was no difference in health care utilization between the treatment arms, so out-of-pocket costs and productivity losses were the same for the treatment groups (Table 4). In the third scenario, where we extrapolated the effects of the iron interventions beyond trial follow-up, iron supplements cost $817 (95% CI: $648, $1109) per DALY averted (Supplemental Table 4). In all 3 scenarios, MNPs remained dominated by iron supplementation (that is, more costly and less effective).

**FIGURE 2:**
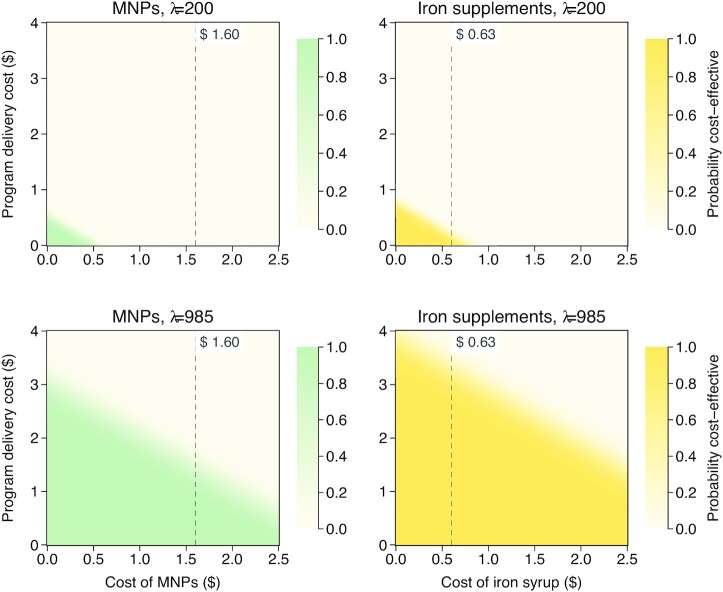
Two-way sensitivity analysis of impact of changes in active iron agents and program delivery costs on the probability of cost-effectiveness. Contour plots showing the impact of changes in costs of active iron agents (iron syrups or MNPs) and program delivery costs on the probability of cost-effectiveness obtained via *P* values of NMB regression where NMB = λ × Δdisability-adjusted life-years – ΔCosts. The top rows are for a threshold (λ) of $200 whereas the bottom rows are for a threshold of $985 (half the gross domestic product per capita of Bangladesh in 2020). The left column is for MNPs where the dotted lines mark the base case cost of active iron agent ($1.60 for a 3-mo supply of MNPs). The right column is for iron supplements where the dotted lines mark the base case cost of active iron agent ($0.63 for a 3-mo supply of iron syrups). MNP, multiple micronutrient powder; NMB, net monetary benefit; λ, willingness to pay threshold.

In the 1-way sensitivity analysis, the assumptions that made iron supplementation cost <$1500 per DALY averted were using the upper 95% uncertainty interval for anemia disability weight or lowering program delivery cost by 50% (**Supplemental Figure 4**). We then explored what combinations of cost of program delivery and cost of active iron interventions would make either iron supplements or MNPs optimal with ≥50% probability (**Figure 2**). At a threshold ceiling of $200 per DALY averted, neither intervention was cost-effective at any program delivery cost. At $985 (half GDP per capita), the interventions would have ≥50% probability of being cost-effective if program delivery costs were <$1.50/child (for MNPs) or <$3.50/child (for iron supplements). These are well below the base case program delivery costs of $5.80 (in 2020 prices). **Supplemental Figure 5** presents a 2-way sensitivity analysis at $1970 (GDP per capita).

Finally, under very optimistic scenarios, our threshold sensitivity analysis suggests program delivery costs must be <$5.60/child for iron supplements to have ≥50% probability of being cost-effective (**Supplemental Figure 6**). In comparison, the base case cost was $5.80/child. This result represents a best-case scenario—the adherence in the complete cases data was >80%, we used a threshold ($985) higher than empirical estimates of health opportunity costs in Bangladesh, and we assumed sustained benefits of iron beyond the trial follow-up.

## Discussion

This study investigated the cost-effectiveness of universal iron supplementation or provision of MNPs compared with no intervention in children younger than 2 y of age in Bangladesh. To our knowledge, this is the first study using robust data from a randomized controlled trial rather than observational data, and thus our findings offer important insights into the expected value for money of iron interventions in other non-malaria-endemic settings. Our findings show that neither intervention was cost-effective at cost-effectiveness thresholds of $200 and $985 (half GDP per capita of Bangladesh in 2020). The probability that MNPs are cost-effective was 0% at any cost-effectiveness threshold. If the threshold were raised to 1 GDP per capita for Bangladesh ($1970), then iron supplementation would be cost-effective; however, this threshold is likely to be unrealistic for many low- and middle-income countries (LMICs) (53). Our conclusions remained robust to various sensitivity and scenario analyses; specifically, program delivery costs would need to be very low to render these interventions cost-effective.

The BRISC trial was the first randomized controlled trial with long enough follow-up to have the potential to show a substantial difference in cognitive development outcomes; however, no differences in cognitive scores or reports of diarrhea and other infections were observed between the treatment groups (20). Accordingly, we did not include the effects of iron supplements or MNPs on cognitive development in the cost-effectiveness analysis. Previous studies that reported that MNPs were good value for money used averted productivity losses from improved cognition as a health outcome, imputed from observational studies or non-empiric estimates (23, 24, 28). However, both the BRISC trial (20) and previous systematic reviews identified no effect of both interventions on cognition in children aged 6–23 mo (13, 14). Our findings indicate the value of using empiric data to justify effect sizes for key outcomes in economic analyses.

Two strengths of this study were using data from a randomized clinical trial with a large sample size and the use of advanced statistical methods to capture uncertainty. The sample size was large enough to detect slight differences in functional outcomes, hemoglobin concentrations, and diarrheal episodes between the groups. Missing data on hemoglobin concentration were handled using multiple imputation. Adherence data were complete, enabling this variable to be used in the imputation model with higher accuracy for the values generated. By combining imputation with nonparametric bootstrapping, the analysis captured the uncertainty surrounding the ICER in the presence of missing data.

This study obtained costs of iron supplements and MNPs from local manufacturers and then explored the impact of varying cost parameters on overall cost-effectiveness. The price per 90 sachets of MNP used was $1.60. In comparison, UNICEF supplied the same quantity of MNPs at $1.65 for 3 boxes (90 sachets) per child between 2015 and 2020 (21). Therefore, the costs of MNP used in the BRISC trial reflected global prices. We assumed that either intervention (syrup or MNP) would require a similar intensity of program delivery activities primarily intended to encourage adherence. Nevertheless, we varied the cost of active iron agents (syrups or MNPs) and program delivery in a 2-way sensitivity analysis. Our results showed that, using a threshold of 0.5 × GDP per capita and cost of iron syrups at $0.63/child, program delivery costs will need to be as low as $3.50 · child^−1^ · y^−1^ in 2020 prices for iron supplementation to be cost-effective. In a very optimistic scenario, the minimum program delivery costs for cost-effectiveness were $3.25 for MNPs and $5.60 for iron supplements. Comparing with costs reported in pilot programs (22, 34), keeping program delivery costs below these amounts without affecting adherence, coverage, and ultimately DALYs averted could be difficult. One strategy to reduce the cost of delivering MNP programs is to utilize personnel and resources of existing Infant and Young Child Feeding interventions (54).

We used 2 reasonable cost-effectiveness thresholds for Bangladesh: $200 and $985 (half GDP per capita in 2020) (48, 49). A 2017 ranking of publicly funded health interventions in LMICs found more than half of interventions had an ICER < $200 per DALY averted (55). Previous studies have estimated the threshold in Bangladesh to be $150 per DALY averted (49) and $230 per quality-adjusted life-year (QALY) gained (48). Half GDP per capita was used because it has been suggested as an appropriate measure of health opportunity costs in LMICs (53). Our results indicate the ICER of $1645 per DALY averted for iron supplements (which dominate MNPs) was well above these decision thresholds. The approach taken to capture the intervention benefits in terms of DALYs averted through reductions in anemia does not incorporate potential benefits of MNPs due to the other micronutrients provided. However, the BRISC trial did not find differential effects between MNPs and iron supplements for outcomes potentially associated with added micronutrients such as corneal lesions (vitamin A), stunting (zinc), and diarrhea (zinc) (20). Therefore, capturing effects of other micronutrients provided is unlikely to change our findings that neither MNPs nor iron supplements are likely to be cost-effective in this rural Bangladeshi setting, and possibly other LMICs where epidemiology of anemia and diarrhea, and health care costs are similar.

Our study included all cost components relevant to estimating the total costs of iron interventions. The BRISC trial collected data on health care utilization in the form of number of unplanned visits; we then used guidelines to guide assumptions on estimating provider costs. Collecting detailed cost data would not have provided information on variation across providers that may have occurred if the intervention had been carried out in a real-world setting because caregivers were encouraged to visit a trial pediatrician when necessary. Our base case analysis used a limited health system perspective, so direct nonmedical costs (such as transportation) borne by households and indirect costs due to lost productivity were excluded. We did not expect that either of these 2 limitations would have an impact on our base case results given there was no meaningful difference in unplanned medical visits due to diarrhea between the groups. Because of data limitations, we opted to use GDP per capita per day to estimate productivity losses. Although this may overestimate the true productivity losses, the overestimation is minimal because of the short time frame of our study and low number of lost hours due to illness from diarrhea. Our scenario analysis using a societal perspective showed comparable results with the base case.

A limitation in this study is that results are only valid for a 1-y time horizon. Extrapolating beyond this horizon could reduce the ICER because, at the end of the follow-up period of the trial, children who had received an iron intervention still had ≥20% reduction in anemia prevalence (20). However, it is unclear how long these benefits will last, so estimating the DALYs averted over an extended period would be problematic. In a scenario analysis where we assumed iron interventions had sustained benefits on anemia for an additional 3 mo, the ICER for iron supplements ($807/DALY averted) compared with no intervention became lower than half of GDP per capita. Another limitation was that we calculated anemia and diarrhea comorbid YLDs using an additive method. Although this is not ideal, it would have a minor impact on results owing to the short nature of diarrhea episodes. Finally, because the trial protocol included weekly visits by VHWs intended to encourage adherence, the results seen in the trial are likely to be more optimal than in real life, leading us to underestimate the calculated ICER.

The WHO recommends universal provision of iron interventions to reduce the prevalence of anemia in children <2 y old. However, with ICERs > $1000 per DALY averted, universal iron interventions do not appear cost-effective in this rural Bangladeshi setting. Iron supplements could become cost-effective under very optimistic scenarios and at program delivery costs <$5.60/child. These findings have important implications for policy and funding decisions for nutrition programs across LMICs. Further research should investigate whether these results hold in other settings, for example, in malaria-endemic areas, and evaluate whether other interventions, such as iron supplementation during pregnancy, could offer solutions to reduce the impact of anemia in young children.

## Supplementary Material

nqac225_Supplemental_File

## Data Availability

Data described in the article, code book, and analytic code will be made available upon request to the Senior Authors pending application and approval.

## Notes

Supported by Australian National Health and Medical Research Council grants GNT1103262 (to S-RP and B-AB), GNT1159151 (to S-RP), and GNT1158696 (to S-RP). icddr,b is grateful to the Government of Bangladesh, Global Affairs Canada (GAC), the Swedish International Development Cooperation Agency (Sida), and the Foreign, Commonwealth, and Development Office (FCDO) United Kingdom for providing core/unrestricted support. We thank Renata Ltd. for provision of the multiple micronutrients powder/placebo.

This work was supported by the Australian National Health and Medical Research Council GNT1103262, GNT1159151, GNT1158696 and GNT2009047. icddr,b is grateful to the Governments of Bangladesh, Canada, Sweden and the UK for providing core/unrestricted support. We thank the local field workers, participants and their families for their involvement in the study.

BA-B and NC report grants from National Health and Medical Research Council (NHMRC), during the conduct of the study. S-RP reports grants from NHMRC, during the conduct of the study; grants from Bill and Melinda Gates Foundation, personal fees from Vifor Pharma, personal fees from Keros Pharma, outside the submitted work. All other authors declare no conflict of interest.

The study sponsors had no role in study design, data collection, analysis and interpretation, report writing, and the decision to submit the paper for publication.

Supplemental Methods, Supplemental Figures 1–6, and Supplemental Tables 1–4 are available from the “Supplementary data” link in the online posting of the article and from the same link in the online table of contents at https://academic.oup.com/ajcn/.

EA and SJH contributed equally to this work as joint first authors. S-RP and NC contributed equally to this work as joint senior authors.

Abbreviations used: BRISC, Benefits and Risks of Iron Interventions in Children; CEAC, cost-effectiveness acceptability curve; DALY, disability-adjusted life-year; GDP, gross domestic product; ICER, incremental cost-effectiveness ratio; LMIC, low- and middle-income country; MNP, multiple micronutrient powder; NMB, net monetary benefit; ORT, oral rehydration therapy; VHW, village health worker; YLD, year lost due to disability; YLL, year of life lost.
