# Supplementary material for: Cost-effectiveness of universal iron supplementation and iron-containing micronutrient powders for anemia among young children in rural Bangladesh: analysis of a randomized, placebo-controlled trial
Source: Am J Clin Nutr. 2022 Oct 3;116(5):1303–13. doi: 10.1093/ajcn/nqac225 (PMC9630870; doi:10.1093/ajcn/nqac225)
Supplement: nqac225_Supplemental_File [file nqac225_supplemental_file.docx]

Supplementary material – Cost-effectiveness of universal iron supplementation and iron-containing micronutrient powders for anemia among young children in rural Bangladesh: analysis of a randomised, placebo-controlled trial

[Supplementary Methods 2](#_Toc105631374)

[Search Terms for Literature Review 2](#_Toc105631375)

[CONSORT Flow Diagram 3](#_Toc105631376)

[Disability weights and years of life lived with disability (YLD) calculations 4](#_Toc105631377)

[Missing data 6](#_Toc105631378)

[Estimating costs outside the health system 7](#_Toc105631379)

[Supplementary Results 8](#_Toc105631380)

[References for supplementary material 17](#_Toc105631381)

# Supplementary Methods

## Search Terms for Literature Review

CABDirect Global Health (1973 to 25 April 2021) searched on 27 April 2021

((anaemia OR anemia) AND (iron OR ("micronutrient powder*")) AND (child* OR infant*) AND (("economic evaluation") OR ("cost effective*") OR DALY*)) AND ( ((sc:(( "HE" ) )) ))

Ovid MEDLINE (1946 to 23 April 2021) searched on 27 April 2021

((anaemia or anemia) and (iron or "micronutrient powder*") and (child* or infant*) and ("economic evaluation" or "cost effective*" or DALY*)).af.

## CONSORT Flow Diagram

Supplementary Figure 1. CONSORT Flow Diagram for BRISC Trial

## Disability weights and years of life lived with disability (YLD) calculations

Thresholds for hemoglobin concentrations to diagnose anemia according to WHO guidelines[^1^](#_ENREF_1) are given in Supplementary Table 1. The table also includes the corresponding disability weights for the severity levels of anemia, which were obtained from Global Burden of Disease 2019.[^2^](#_ENREF_2)

Supplementary Table 1. Hemoglobin levels to assess severity of anemia

| Hemoglobin (grams/Litre) | Diagnosis* | Disability weight† |
| --- | --- | --- |
| 110 or higher | None | 0.000 |
| 100 to 109 | Mild anemia | 0.004 (95% CI 0.001, 0.008) |
| 70 to 99 | Moderate anemia | 0.052 (95% CI 0.034, 0.076) |
| 70 or lower | Severe anemia | 0.149 (95% CI 0.101, 0.209) |

* WHO cut-offs Hemoglobin concentrations for diagnosing and assessing severity of anemia[^1^](#_ENREF_1)

† Magnitude of health loss associated with various severity of anemia from GBD 2019 [^2^](#_ENREF_2)

Anemia was assessed using capillary hemoglobin (Hb) levels measured by HemoCue-301^®^. Hemoglobin levels were measured at baseline, at the end of the intervention period and at the end of the follow-up period. The YLDs due to anemia were an average of disability weights for the appropriate severity of anemia weighted by the duration of time a child was in the particular (non) anemic state. It is given as.

$$YLD=t_{int}*\frac{{DW}_{0}+{DW}_{3}}{2}+t_{post}*\frac{{DW}_{3}+{DW}_{12}}{2}$$

Where ${DW}_{0}$ refers to disability weight for anemia status at randomization (0 months). ${DW}_{3}$ refers to disability weight for anemia status at 3 months from randomization (end of intervention). ${DW}_{12}$ refers to disability weight for anemia status at 12 months from randomization (9 months of post-intervention follow-up). $t_{int}$ refers to proportion of time spent in intervention phase. $t_{int}=\left( \frac{3 months}{12 months} \right)=0.25$. $t_{post}$ refers to proportion of time spent in intervention phase. $t_{post}=\left( \frac{9 months}{12 months} \right)=0.75$. For example, if a child was healthy (not anemic) at baseline, had mild anemia after 3 months and severe anemia at 12 months, then $t_{int}=0.25$; $t_{int}=0.75$; ${DW}_{0}$ = 0.004; ${DW}_{3}$ = 0.149; ${DW}_{12}$ = 0.000, and the disability weight is

$$YLD=0.25*\frac{0.000+0.004}{2}+0.75*\frac{0.004+0.149}{2}=0.058$$

Morbidity from diarrhea was captured weekly during the intervention period and monthly during the follow-up period. Cases were categorized as moderate (if they treated in outpatient departments) or severe (if they were hospitalized) with duration of illness of 6.4 and 8.4 days, respectively.[^3^](#_ENREF_3) However, the GBD uses clinical descriptors of diarrhea which may not be directly reflected by our categorization

- Moderate diarrheal diseases (disability weight 0.188, 95% uncertainty interval [UI] 0.125, 0.264): has diarrhea three or more times a day, with painful cramps in the belly and feeling thirsty.
- Severe diarrheal diseases (disability weight 0.247, 95% UI 0.164, 0.348): has diarrhea three or more times a day with severe belly cramps. The person is very thirsty and feels nauseous and tired.

For children with anemia who experienced diarrhea, we used an additive approach to account for joint disability due to anemia and diarrhea comorbidity. A drawback of using an additive method is the joint disability may exceed 1.0, but there is negligible risk of that in our study.

## Missing data

Complete data for hemoglobin level was available for 46% of participants (N=3300). 24% had baseline data only, and 30% had incomplete data. Cases with missing baseline data (n=112) were dropped from the analyses. For the base case analysis, missing hemoglobin data were calculated using multiple imputation by chained equations (MICE), stratified by treatment group, with separate model at each study visit.[^4^](#_ENREF_4) Data were assumed to be missing at random (MAR), that is, given our data the probability of missingness does not depend on the unobserved values. The number of imputed data sets was greater than the percentage of missing hemoglobin data within any treatment group.[^5^](#_ENREF_5) The highest proportion of missing data was observed among placebo group at the post-intervention visit (39.8% missing values); therefore 40 imputations were performed. The imputation model included union, sex of the child, family care indicator (FCI) score, maternal education, and 70% adherence as covariates. Missing data in these covariates were mean imputed before including in the multiple imputation model. This was done by replacing missing values using the pooled mean of non-missing data from all treatment groups.[^6^](#_ENREF_6)

We did not adjust for missing diarrhea data in the analyses. There were 1102 clinic visits during the intervention period and 1531 visits during the follow-up period. The reason for healthcare visits was collected for 67% of all visits during the intervention and for 95% of the trial population during the follow-up period. Missing diarrhea data was mainly due to change of data collection form during the trial – this implies missing data was independent from treatment allocation and expected to occur similarly across study arms due to the randomised block design which balances the study arms every three or six participants. Therefore, we assumed that participants for whom diarrhea data was not collected did not behave different between the treatment arms compared to those for whom we have their data available.

## Estimating costs outside the health system

Out-of-pocket expenditure amounts and number of hours off work by caregivers when their child had diarrhea was collected in the BRISC trial. Direct medical out-of-pocket expenditures (medications, tests, consultation fees) were dropped because this would be covered in cost of diarrhea treatment to be borne by the healthcare system, dropping them avoids situations where richer caregivers can afford more expensive medications, and some of these visits were not due to diarrhea only, so these expenses may be due to other non-related illness. Therefore, out-of-pocket expenditure amounts were based on direct non-medical costs only. These non-medical costs included amounts spent on transportation to medical provider, food and accommodation, and other non-medical expenses directly related to the child’s visit or stay in the hospital.

Indirect costs included productivity losses by caregivers due to diarrhea by a child, calculated using the human capital approach (HCA). In HCA, any hour not worked is counted as an hour lost and this duration of absence is valued by the achievable income.[^7^](#_ENREF_7) Caregivers reported the length of time that they had to stop doing usual work to care for their child who had diarrhea and visited the hospital or was hospitalized. The number of hours reported at the initial and recurrent visits were summed, divided by the number of waking hours (12 hours), and multiplied by the GDP per capita per day for 2020 to calculate the total indirect costs of illness. We applied this calculation whether the usual activities were paid or unpaid.

## Program delivery costs

It was not feasible to disentangling trial protocol-driven costs to obtain program delivery costs, so we used estimates from literature. The BRAC sales intervention in Bangladesh estimated five-year program delivery cost of $14 million but did not calculate costs per child. [^8^](#_ENREF_8)^,^[^9^](#_ENREF_9) The HFTAG estimated a cost of $4.50 in 2013 ($5.80 in 2020 prices).[^10^](#_ENREF_10) More recently, The Strengthening Partnerships, Results, and Innovations in Nutrition Globally (SPRING) project piloted nine months of MNP distribution in Uganda at an estimated program cost delivery cost of $53 per child.[^11^](#_ENREF_11) We considered SPRING estimates extremely high. The HFTAG estimates have been used in a previous study on cost-effectiveness of micronutrient powders.[^12^](#_ENREF_12) Also, dividing BRAC costs by the total number of children living in the study area (5.4 million) and adjusting by reach data from the program (about 50%) yields a cost per child of $5.40. Therefore, our base case used HFTAG estimates. We did not find confidence intervals on these costs so could not fully explore the uncertainty in costs.

Supplementary Table 2. Characteristics of participants by treatment group and availability of Hemoglobin data^1^

| Variable | Placebo | | MNPs (fortification) | | Iron supplements | |
| --- | --- | --- | --- | --- | --- | --- |
|  | Missing (N=609) | Complete (N=491) | Missing (N=593) | Complete (N=506) | Missing (N=589) | Complete (N=512) |
| Female sex | 291 (47.8%) | 259 (52.7%) | 301 (50.8%) | 247 (48.8%) | 273 (46.3%) | 277 (54.1%) |
| Wealth index | -0.0 (2.4) | -0.1 (2.5) | 0.1 (2.4) | -0.1 (2.4) | 0.1 (2.4) | -0.2 (2.5) |
| Family care indicator^2^ | 13.6 (7.2) | 13.7 (6.9) | 13.3 (7.2) | 13.3 (7.1) | 13.2 (6.9) | 13.3 (7.3) |
| Maternal education |  |  |  |  |  |  |
| No education | 29 ( 4.8%) | 20 ( 4.1%) | 27 ( 4.6%) | 16 ( 3.2%) | 21 ( 3.6%) | 32 ( 6.3%) |
| 1-8 years | 306 (50.2%) | 261 (53.2%) | 316 (53.3%) | 287 (56.7%) | 324 (55.0%) | 267 (52.1%) |
| 9-12 years | 254 (41.7%) | 193 (39.3%) | 227 (38.3%) | 193 (38.1%) | 223 (37.9%) | 198 (38.7%) |
| >12 years | 20 ( 3.3%) | 17 ( 3.5%) | 23 ( 3.9%) | 10 ( 2.0%) | 21 ( 3.6%) | 15 ( 2.9%) |
| Union |  |  |  |  |  |  |
| Bhulta | 174 (28.6%) | 185 (37.7%) | 179 (30.2%) | 181 (35.8%) | 190 (32.3%) | 170 (33.2%) |
| Golakandail | 244 (40.1%) | 143 (29.1%) | 234 (39.5%) | 153 (30.2%) | 240 (40.7%) | 150 (29.3%) |
| Rupganj | 191 (31.4%) | 163 (33.2%) | 180 (30.4%) | 172 (34.0%) | 159 (27.0%) | 192 (37.5%) |
| Baseline anemia^3^ | 254/576 (44.1%) | 219/491 (44.6%) | 224/551 (40.7%) | 239/506 (47.2%) | 260/561 (46.3%) | 236/512 (46.1%) |
| Baseline iron deficiency^4^ | 152/548 (27.7%) | 120/482 (24.9%) | 144/531 (27.1%) | 130/494 (26.3%) | 167/536 (31.2%) | 140/498 (28.1%) |
| Baseline iron deficiency anemia^5^ | 104/548 (19.0%) | 84/482 (17.4%) | 92/531 (17.3%) | 90/494 (18.2%) | 122/536 (22.8%) | 103/498 (20.7%) |
| 70% Adherence^6^ | 402 (66.0%) | 410 (83.5%) | 362 (61.0%) | 416 (82.2%) | 354 (60.1%) | 421 (82.2%) |

^1^ Data are presented as mean (SD) for continuous measures, and n (%) or n/total (%) for categorical measures.

^2^ Scores on family care indicator total score range from 0 to 42, with higher scores indicating more activities.

^3^ Anemia defined as hemoglobin level of <11 g per decilitre).

^4^ Iron deficiency was defined as a ferritin level of <12 *μ*g per litre or <30 *μ*g per litre if the C-reactive protein level was >5 mg per litre).

^5^ Iron deficiency anemia was defined as concurrent anemia and iron deficiency.

^6^ Proportion of children that consumed at least 70% of their assigned active iron agents or placebo.

Supplementary Table 3. Cost-effectiveness results for one-way comparisons of no intervention, MNPs, and iron supplements^1^

|  | **No intervention**  **Mean (SD)** | **MNPs**  **Mean (SD)** | **Iron supplements**  **Mean (SD)** | **MNPs vs no intervention**  **Mean (95% CI)^2^** | **Iron supplements vs no intervention**  **Mean (95% CI)^2^** | **Iron supplements vs MNPs**  **Mean (95% CI)^2^** |
| --- | --- | --- | --- | --- | --- | --- |
| Cost and incremental costs | $ 0.62 (2.58) | $ 8.12 (2.94) | $ 7.06 (2.58) | $ 7.50 (7.27 to 7.72) | $ 6.44 (6.22, 6.67) | -1.06 (-1.28, -0.81) |
| DALYs AND DALYs averted (95% CI) | 0.0075 (0.013) | 0.0044 (0.009) | 0.0036 (0.008) | 0.0031 (0.0022 to 0.0041) | 0.0039 (0.0030, 0.0048) | 0.0008 (0.0001, 0.0015) |
| ICER (95% CI) | … | … | … | 2400 (1842, 3441) | 1645 (1333, 2153) | -1337 (-7068, -630) |

^1^ Costs and incremental costs are calculated from health system perspective and expressed in 2020 US Dollars ($).

^2^ 95% Confidence intervals (CI) were calculated via bias-corrected and accelerated bootstrapping (two thousand replications on multiply imputed data). MNPs – micronutrient powders; DALYs – disability-adjusted life-years; ICER – incremental cost-effectiveness ratio.


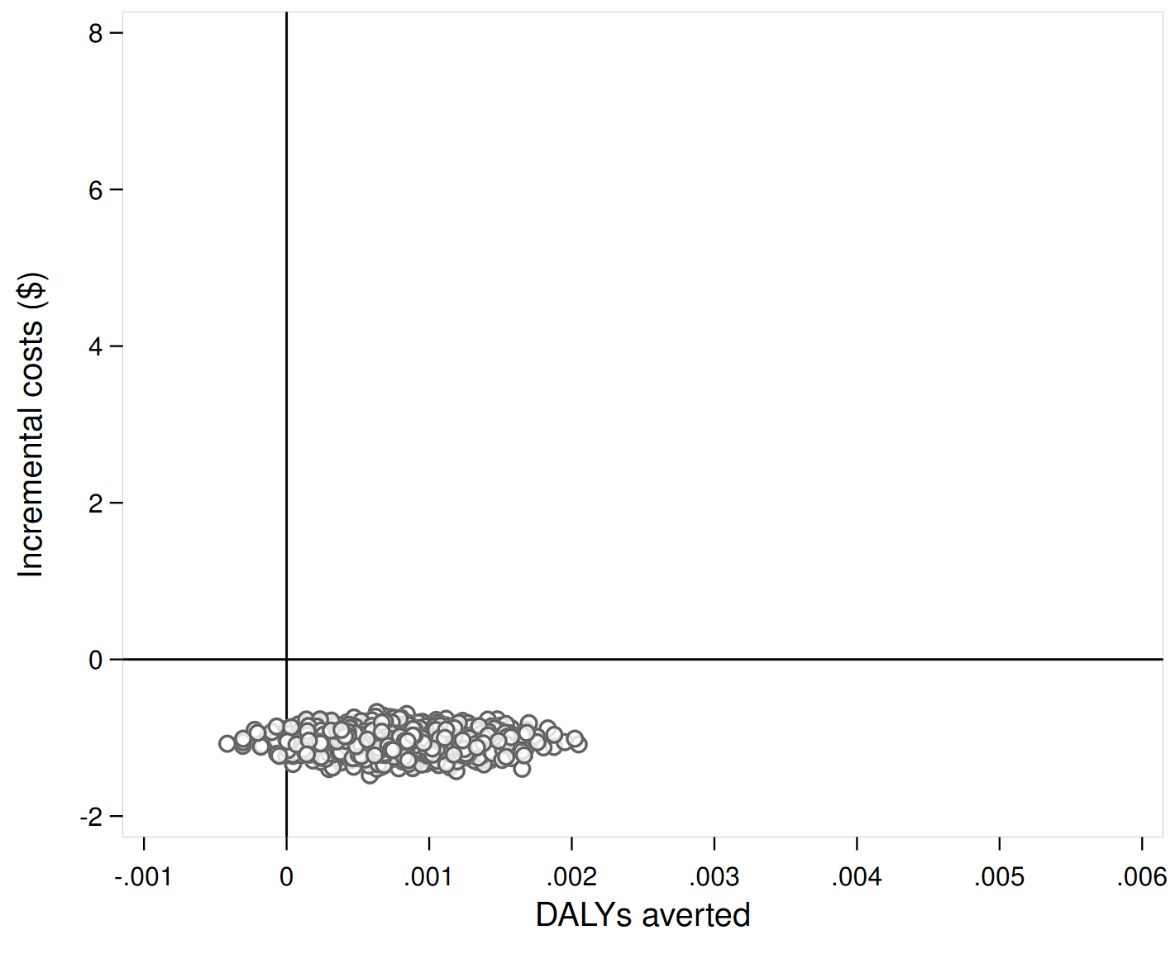
Supplementary Figure 2. Incremental cost-effectiveness plane for Iron supplementation vs MNPs


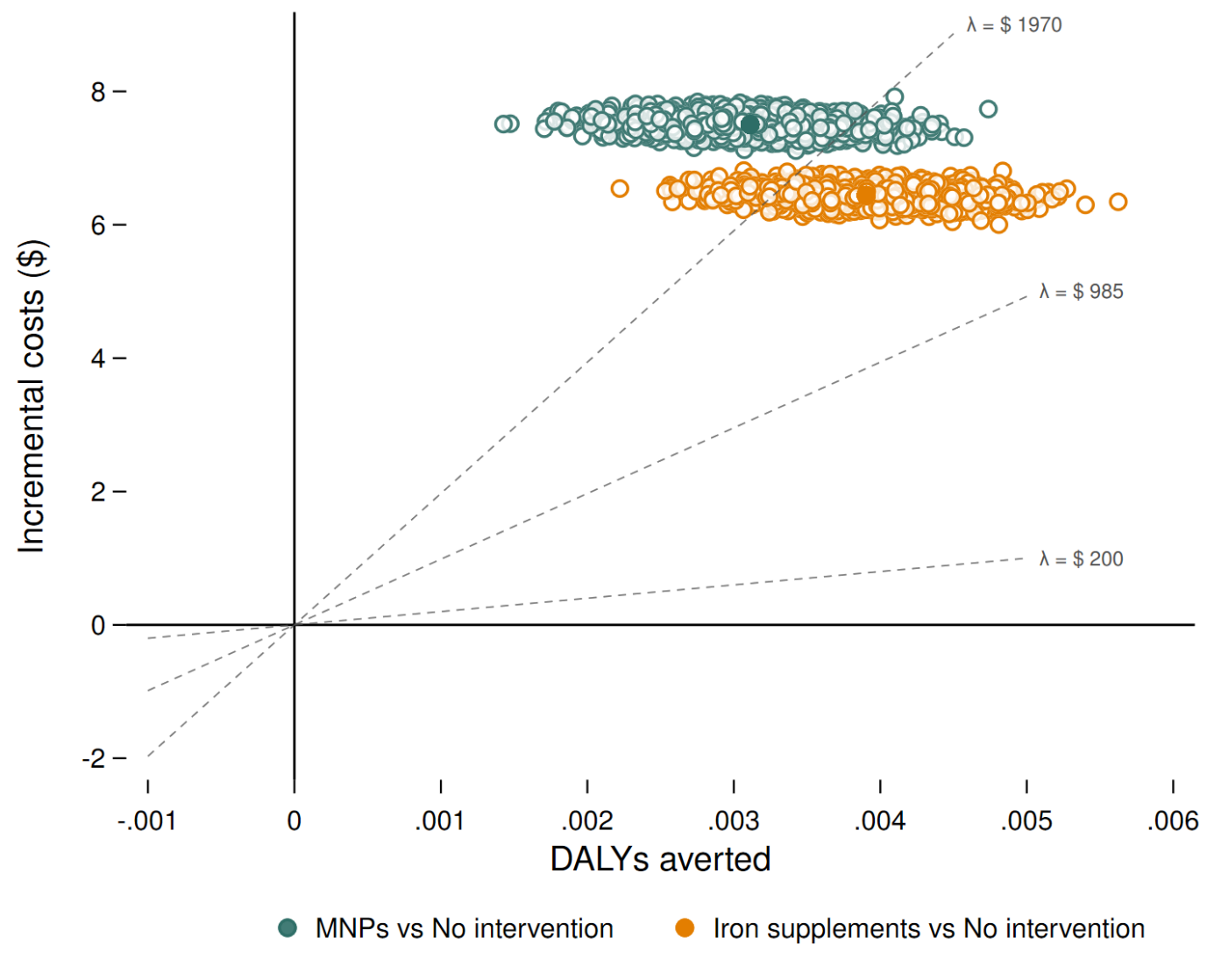


Supplementary Figure 3. Cost-effectiveness plane showing joint distribution of incremental costs and DALYs from two thousand bootstrapped replications from multiply imputed study data.

Each open-coloured circle in the scatterplot represents one bootstrap resample. The graph shows iron supplements versus no intervention (orange circles) and MNPs versus no intervention (green circles). The filled circles represent the point estimate of incremental cost-effectiveness ratio. Because these replicates lie in the northeast quadrant of the plane, the interventions (MNPs and iron supplements) are more effective but also more costly than having no intervention, implying the cost-effectiveness depends on cost-effectiveness thresholds.

MNPs – micronutrient powders; DALYs – disability-adjusted life-years’ GDP – gross domestic product; λ – cost-effectiveness threshold

Supplementary Table 4. Results of scenario analyses per child (with bootstrapped 95% confidence intervals) comparing iron intervention strategies^1^

| **Strategy** | **Mean cost (SD)** | **Incremental cost (95% CI)** | **Mean DALYs (SD)** | **DALYs averted (95% CI)** | **ICER (95% CI)** |
| --- | --- | --- | --- | --- | --- |
| *Scenario 1: complete cases only* |  |  |  |  |  |
| No intervention | $ 0.69 (2.64) | reference | 0.0110 (0.016) | reference | reference |
| MNPs | $ 7.83 (1.82) | $ 7.14 (7.98, 7.30) | 0.0063 (0.011) | 0.0047 (0.0030, 0.0063) | dominated by iron supplements^2^ |
| Iron supplements | $ 7.07 (2.60) | $ 6.39 (6.21, 6.56) | 0.0047 (0.009) | 0.0063 (0.0048, 0.0079) | $ 1011 (802, 1350) |
| *Scenario 2: societal perspective* |  |  |  |  |  |
| No intervention | 1.78 (8.04) | reference | 0.0075 (0.013) | reference | reference |
| MNPs | 9.27 (7.24) | $ 7.51 (6.87, 8.11) | 0.0044 (0.009) | 0.0031 (0.0022, 0.0041) | dominated by iron supplements^2^ |
| Iron supplements | 8.50 (9.70) | $ 6.74 (0.00, 7.53) | 0.0036 (0.008) | 0.0039 (0.0030, 0.0048) | $ 1720 (1338, 2292) |
| *Scenario 3: complete cases with extrapolated effects* |  |  |  |  |  |
| No intervention | $ 0.69 (2.64) | reference | 0.0136 (0.020) | reference | reference |
| MNPs | $ 7.83 (1.82) | $ 7.14 (7.98, 7.30) | 0.0081 (0.015) | 0.0055 (0.0034, 0.0077) | dominated by iron supplements^2^ |
| Iron supplements | $ 7.07 (2.60) | $ 6.39 (6.21, 6.56) | 0.0058 (0.012) | 0.0078 (0.0058, 0.0098) | $ 817 (648, 1109) |

^1^ Costs and incremental costs are expressed in 2020 United States dollars ($). 95% Confidence intervals (CI) were calculated via bias-corrected and accelerated bootstrapping (two thousand replications). Scenarios 1 and 3 use participants with non-missing hemoglobin data at all timepoints while scenario 2 uses multiply imputed data.

^2^ The ICERs comparing MNPs with no intervention were not reported in the table because MNPs are dominated by iron supplements in all scenarios. MNPs – micronutrient powders; DALYs – disability-adjusted life-years; ICER – incremental cost-effectiveness ratio.


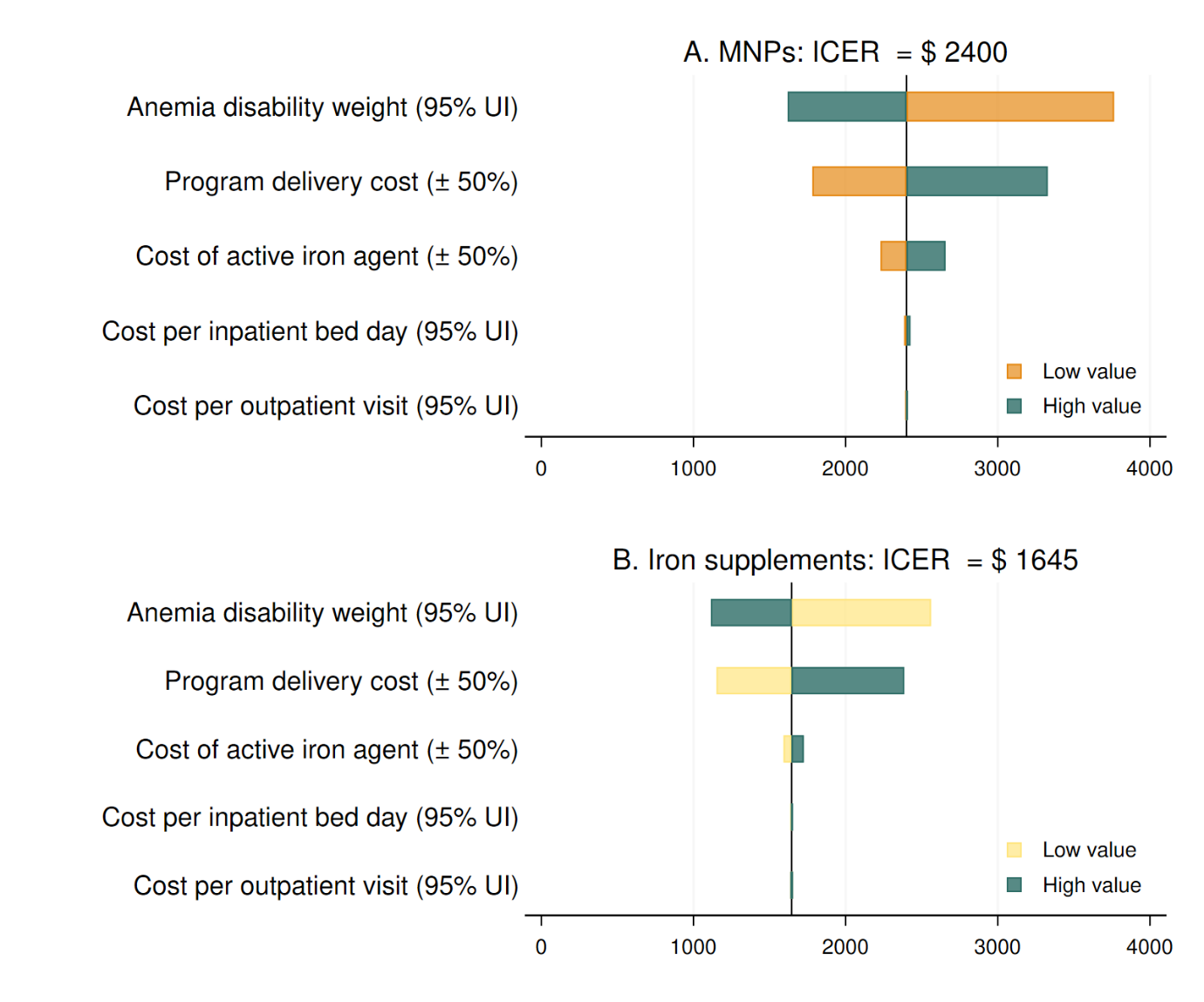


Supplementary Figure 4. One-way sensitivity analysis showing impact of changes in input values on ICER.

Impact of using the low and high values on the resulting incremental cost-effectiveness ratio. The active iron agent is in form of powders for MNP fortification (A) and syrup for iron supplements (B) strategies. MNPs – micronutrient powders; DALYs – disability-adjusted life-years; ICER – incremental cost-effectiveness ratio; UI – uncertainty interval


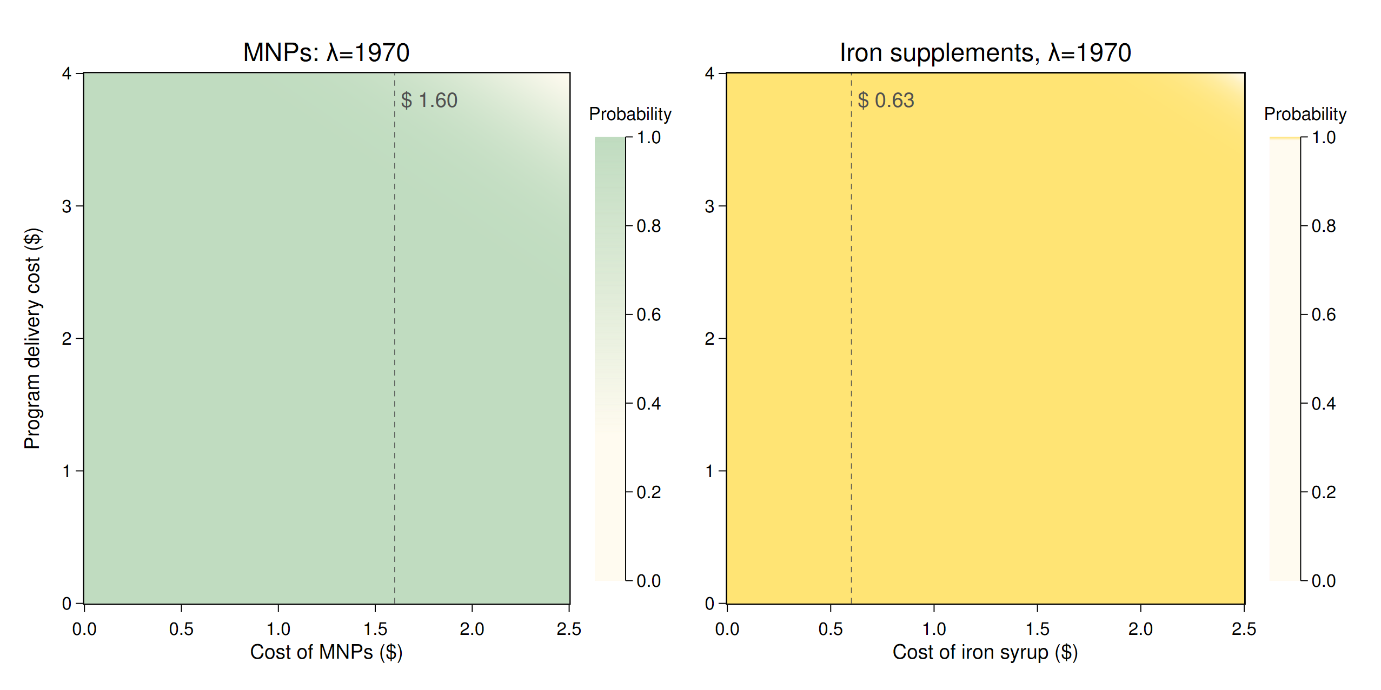


Supplementary Figure 5. Two-way sensitivity analysis impact of changes in active iron agents and program delivery costs on the probability of cost-effectiveness when the threshold is GDP per capita


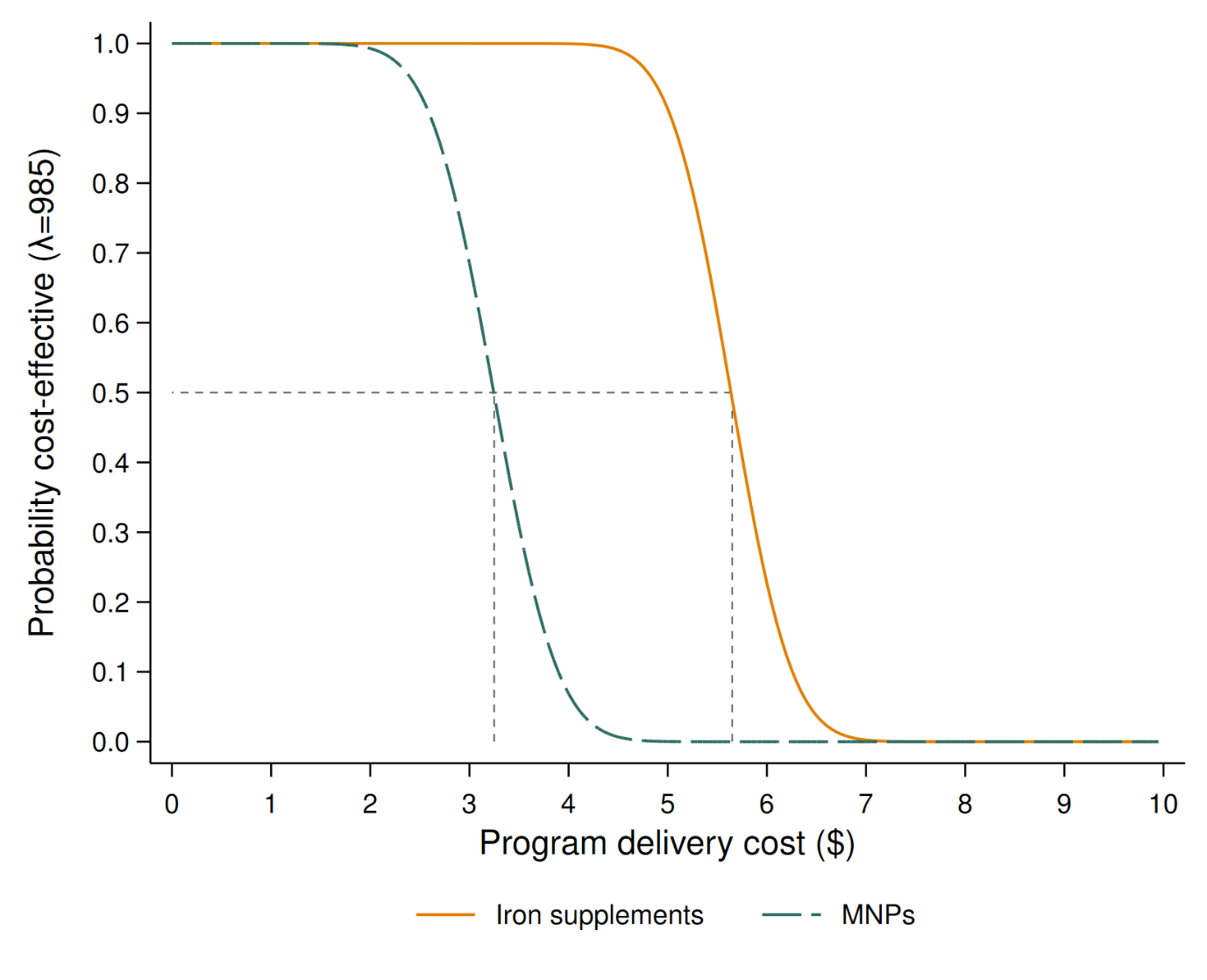


Supplementary Figure 6. Threshold sensitivity analysis on impact of change in program delivery costs on the probability of cost-effectiveness under very optimistic scenarios

The probability of cost-effectiveness was obtained via p-values of NMB regression where NMB=λ× ΔDALYs –ΔCosts. The dotted lines illustrate that program delivery costs will need to be below $5.60 per child for iron supplements ($3.25 per child for MNPs) to have at least 50% probability of being cost-effective (the base case cost is $5.80 per child). MNPs – micronutrient powders; NMB – net monetary benefit; λ – cost-effectiveness threshold

# References for supplementary material

1 World Health Organization. Hemoglobin concentrations for the diagnosis of anemia and assessment of severity. Geneva: World Health Organization, 2011.

2 Global Burden of Disease Collaborative Network. Global Burden of Disease Study 2019 (GBD 2019) Disability Weights. . Seattle, USA: Institute for Health Metrics and Evaluation (IHME); 2020.

3 Lamberti LM, Fischer Walker CL, Black RE. Systematic review of diarrhea duration and severity in children and adults in low- and middle-income countries. *BMC Public Health* 2012; **12**(1): 276.

4 Braat S, Larson L, Simpson J, et al. The Benefits and Risks of Iron interventionS in Children (BRISC) trial: Statistical analysis plan. F1000Research; 2020.

5 White IR, Royston P, Wood AM. Multiple imputation using chained equations: Issues and guidance for practice. *Statistics in Medicine* 2011; **30**(4): 377-99.

6 Faria R, Gomes M, Epstein D, White IR. A Guide to Handling Missing Data in Cost-Effectiveness Analysis Conducted Within Randomised Controlled Trials. *PharmacoEconomics* 2014; **32**(12): 1157-70.

7 Human Capital Approach. In: Kirch W, ed. Encyclopedia of Public Health. Dordrecht: Springer Netherlands; 2008: 697-8.

8 Ahmed S, Sarma H, Hasan Z, et al. Cost-effectiveness of a market-based home fortification of food with micronutrient powder program in Bangladesh. *Public health nutrition* 2021; **24**(S1): s59-s70.

9 Sarma H, Mbuya MNN, Tariqujjaman M, et al. Role of home visits by volunteer community health workers: to improve the coverage of micronutrient powders in rural Bangladesh. *Public Health Nutrition* 2021; **24**(S1): s48-s58.

10 De Pee S, Flores-Ayala R, Van Hees J, et al., editors. Home Fortification with Micronutrient Powders (MNP). Basel, Switzerland: Home Fortification Technical Advisory Group; 2013.

11 Schott W, Richardson B, Baker E, D'Agostino A, Namaste S, Vosti SA. Comparing costs and cost-efficiency of platforms for micronutrient powder (MNP) delivery to children in rural Uganda. *Annals of the New York Academy of Sciences* 2021; **1502**(1).

12 Pasricha SR, Gheorghe A, Sakr-Ashour F, et al. Net benefit and cost-effectiveness of universal iron-containing multiple micronutrient powders for young children in 78 countries: a microsimulation study. *Lancet Global Health* 2020; **8**(8): e1071-e80.
